# *De novo* Transcriptome Analysis Reveals Distinct Defense Mechanisms by Young and Mature Leaves of *Hevea brasiliensis* (Para Rubber Tree)

**DOI:** 10.1038/srep33151

**Published:** 2016-09-13

**Authors:** Yongjun Fang, Hailiang Mei, Binhui Zhou, Xiaohu Xiao, Meng Yang, Yacheng Huang, Xiangyu Long, Songnian Hu, Chaorong Tang

**Affiliations:** 1 Rubber Research Institute, Chinese Academy of Tropical Agricultural Sciences, Danzhou 571737, Hainan, China; 2 CAS Key Laboratory of Genome Sciences and Information, Beijing Institute of Genomics, Chinese Academy of Sciences, Beijing 100101, China; 3 University of Chinese Academy of Sciences, Beijing 100049, China

## Abstract

Along with changes in morphology in the course of maturation, leaves of *Hevea brasiliensis* become more resistant to leaf diseases, including the South American Leaf Blight (SALB), a devastating fungal disease of this economically important tree species. To understand the underlying mechanisms of this defense, and to identify the candidate genes involved, we sequenced the *Hevea* leaf transcriptome at four developmental stages (I to IV) by Illumina sequencing. A total of 62.6 million high-quality reads were generated, and assembled into 98,796 unique transcripts. We identified 3,905 differentially expressed genes implicated in leaf development, 67.8% (2,651) of which were during the transition to leaf maturation. The genes involved in cyanogenic metabolism, lignin and anthocyanin biosynthesis were noteworthy for their distinct patterns of expression between developing leaves (stages I to III) and mature leaves (stage IV), and the correlation with the change in resistance to SALB and the *Oidium/Colletotrichum* leaf fall. The results provide a first profile of the molecular events that relate to the dynamics of leaf morphology and defense strategies during *Hevea* leaf development. This dataset is beneficial to devising strategies to engineer resistance to leaf diseases as well as other in-depth studies in *Hevea* tree.

*Hevea brasiliensis* (hereafter *Hevea*), cultivated in the tropics and subtropics, is the only commercial source of natural rubber, an important industrial raw material. *Hevea* productivity is influenced by canopy density and photosynthetic efficiency of its leaves. As a shade-tolerant tropical tree species, *Hevea* leaves are exposed to destruction by herbivores when its leaves are tender and expanding. Rubber production and growth of the tree also suffer severely from attack during leaf expansion by various fungal pathogens. Of these, the most devastating leaf pathogen is *Microcyclus ulei* (South American leaf blight, SALB)^1^ that is mainly responsible for the severe problems facing plantation-scale cultivation in Central and South America to which it is endemic and currently confined. The *Hevea* cultivars that contain the highest leaf cyanide potential are reported to have the highest yield potential, suggesting that cyanogenic glucosides act both as defensive chemicals and as an important nitrogen/carbon source^2^. It is hence important to understand the molecular control of chemically defensive metabolites during *Hevea* leaf development.

The *Hevea* canopy refoliates mainly after an annual shedding of the leaves although new leaves can also develop at other times of the year. Typically, leaves develop in sequential flushes on new shoots. Following bud burst, the young leaves, rich in anthocyanin, are initially bronze in color. They are limp and hang with their tips downwards. The leaves then begin to harden, turning pale green and the dark green before reaching full maturity. Morphologically, leaf development is divided into four distinct stages, designated A to D^3^. Physiologically, leaves in stages of A, B and C are generally free of lignin and behave as nutrient sinks^4^,^5^, whereas stage D leaves are source leaves with physiological and structural parameters of mature leaves.

Compared to mature leaves, young leaves of *Hevea* tree are vulnerable to herbivores and pathogen attack. The maturation of *Hevea* leaves takes place over a relatively long period (12–20 days) after bud burst^1^, thus putting *Hevea* into the category of ‘defense’ species that exploit effective secondary metabolites to deter herbivore attack^6^. The vacuolar content of cyanogenic glucosides in *Hevea*, with linamarin as the dominant form, is higher in young leaves than in mature leaves, peaking in green young leaves (stage C)^2^. Cyanogenesis acts as an efficient defense mechanism of young *Hevea* leaves against herbivores, but inhibits active defense reactions against pathogenic diseases^1^,^7^,^8^,^9^, including the SALB. In comparison, *Hevea* mature leaves (stage D) display a decreased cyanogenic ability, but structural hardening and lignin formation act to restrict fungal spread in the cell wall, resulting in complete resistance to SALB. Two types of cytochrome P450 (CYP79D1/D2) and an UDP-glycosyltransferase, as reported in cassava, are responsible for synthesizing linamarin and lotaustralin^8^,^9^,^10^,^11^. Upon tissues being infected and injured, the precursors are set free from the vacuoles and cleaved by linamarase, a β-glycosidase^12^. Subsequently, a hydroxynitrilelyase catalyses the decomposition of in-process product (cyanohydrin) to yield HCN and a carbonyl compound^13^.

It would appear that *Hevea* leaves undergo biochemical and structural changes, especially in the composition of secondary metabolites such as cyanogenic glucosides, anthocyanin, and lignin during the process of development. This contributes to the differing responses of young and mature leaves to biotic and abiotic stresses^1^. However, little is known about the underlying molecular controls.

In this study, we sequenced the transcriptome of *Hevea* leaves in four developmental stages, and generated a panorama of transcriptome dynamics accompanying the leaf development. Investigation of the 3,905 differentially expressed genes identified over the course of leaf development pointed to a number of key genes and networks that affect cyanogenesis, cell wall structure dynamics, and other defensive features. This work would provide essential information for elucidating the combination of chemical and structural defense strategies that protect *Hevea* leaves in their different stages of development. In addition, the transcripts assembled in this study exploit the most in-depth transcriptome sequencing on *Hevea* leaves. The data could serve as a foundation for a myriad of studies on this plant organ.

## Results and Discussion

### Transcriptome sequencing and *de novo* assembly

**Figure 1:**
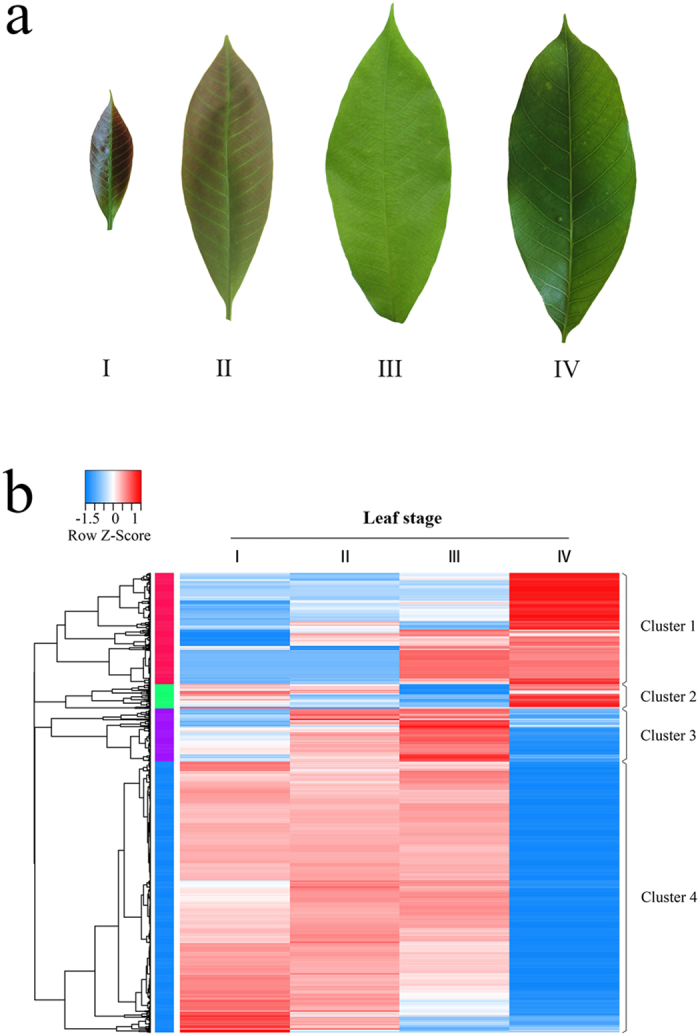
Leaf developmental stages and heat map of differentially expressed genes wherein. (**a**) Four representative stages of *Hevea* leaf development. I, bronze; II, color-change; III, pale-green; IV, mature; (**b**) Expression profile and clustering of 3,095 differentially expressed genes across four developmental leaf stages.

To generate the transcriptome of *Hevea* leaves, cDNA libraries were prepared from four representative stages of leaf development, i.e. bronze (I), color-change (II), pale-green (III) and bright green (mature) (IV) (Fig. 1a), and subjected to paired-end sequencing (2 × 101 bp) using the Illumina HiSeq2000 platform. The broze and color-change leaves correpond to sink leaves, pale-green to the transition from sink to source, and bright green to source. According to the morphological parameters described^3^, the four consecutive leaf stages (I to IV) examined this study correspond to the previously characterized leaf stages of B-C (I to II), C (III) and D (IV), respectively. After filtering adapter sequences and discarding low-quality reads, a total of 62.6 M high-quality paired-end reads with a mean length of 100 bp were attained (Table 1). The sufficiency of RNA-seq reads in each cDNA library was assessed to ensure assembled transcript lengths and number of detected genes were saturated for all the four leaf stages (see Supplementary Fig. S1).

Using the Trinity program^14^, the *de novo* transcriptome assembly yielded 199,472 contigs that included sequence isoforms. To reduce redundancy, only the isoforms that had the highest expression level within each subcomponent were selected. This resulted in a total of 104,137 contigs which were then filtered for trans-self chimeras or trans-multi-gene chimeras^15^, and non-plant proteins. A total of 5341 contigs (338 chimeras and 5003 non-plant proteins) were filtered out. Finally, 98,796 contigs representing unique transcripts with a N50 length of 936 bp (Table 1) served as the mRNA transcript repertoire of *Hevea* leaves.

### Functional annotation and classification

**Figure 2:**
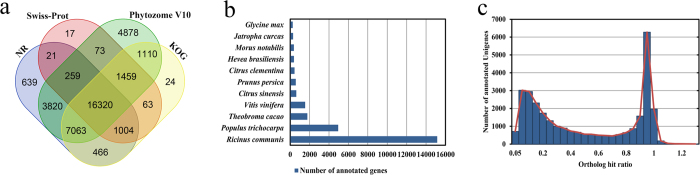
Annotation and assessment of the *Hevea* leaf transcriptome. (**a**) Venn diagram illustrating shared and unique transcripts annotated in databases of Nr, Swiss-Prot, Phytozome and KOG; (**b**) Number of top hits by species from BLASTX results of searches against the Nr database; (**c**) Ortholog hit ratio analysis of the *Hevea* leaf transcriptome assembly.

Of the 98,796 transcripts in the *Hevea* leaf reference transcriptome, 37,216 had at least one hit when matched against the four databases used in this study. Of these, approximately 85% had hits in more than one databases (Fig. 2a). Among the 29,592 genes annotated by the Nr database, 51.2% matched *Ricinus communis* (Fig. 2b), the species that is phylogenetically close to *Hevea*, and 16.7% matched *Populus trichocarpa*. Moreover, 19,724 transcripts assigned to more than one GO term were classified into 44 functional groups of the Gene Ontology database. Using KAAS with eudicots as reference, a total of 5,049 transcripts were mapped to 330 pathways.

To assess the assembly quality of annotated transcripts, we divided the predicted coding length of transcripts by the total coding length of subject genes in the Nr database to compute the ‘ortholog hit ratio’^16^. Among the genes annotated in Nr, about 42% had ratios >0.7, indicating that most genes were fully or almost fully assembled (Fig. 2c). The peak at the ratio of 0.1 might have resulted from incomplete annotation of the short transcripts, ranging from 200 bp to 300 bp.

### Identification of differentially expressed genes

A total of 3,905 differentially expressed genes (DEGs) were identified, of which 196 were between leaf stages I and II (104 up-regulated and 92 down-regulated) (Supplementary Table S1), 415 between leaf stages II and III (264 up-regulated and 151 down-regulated) (Supplementary Table S2). Strikingly, 2,651 were identified between leaf stages III and IV (621 up-regulated and 2,030 down-regulated) (Supplementary Table S3), suggesting that transcriptional regulation plays a critical role in the transition of developing leaves (sink) to mature leaves (source).

Hierarchical clustering cast the 3,905 DEGs across four leaf stages into four distinct clusters (Fig. 1b). Clusters 1 and 4 consisted of the vast majority (74.2%) of the DEGs, and displayed contrasting profiles of gene expression. The Cluster 1 genes were highly expressed in mature leaves whereas the Cluster 4 genes were mainly expressed in developing leaves. To further explore the potential function and metabolic pathway of these two clusters, GO and KEGG enrichment analyses were conducted (Supplementary Table S4). In the 2,056 DEGs of Cluster 4, the GO terms associated with cell structure, division and growth, and the KEGG pathways associated with sugar metabolism and biosynthesis of secondary metabolites were most enriched: this was consistent with the physiological and structural characters of developing *Hevea* leaves^1^. In comparison, the GO groupings and KEGG pathways associated with transport in the 841 DEGs of Cluster 1 were most enriched. This agrees well with the functions of mature *Hevea* leaves as the source organ. Interestingly, thiamine metabolism was also enriched in the Cluster 1 genes, the functions of which remains to be investigated.

### Expression of specific genes in relation to stages of leaf development

**Figure 3:**
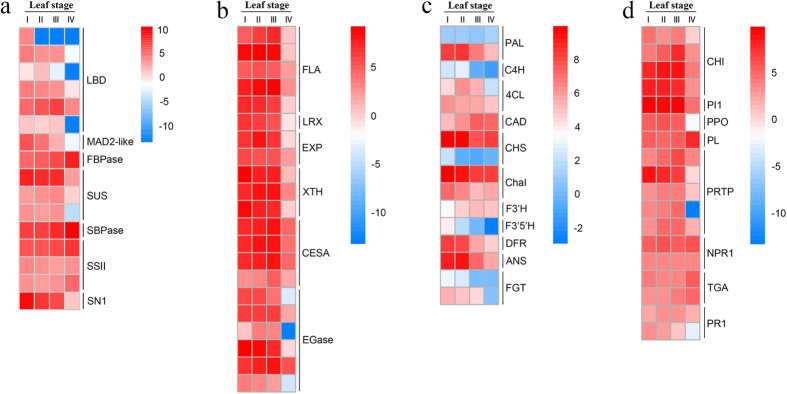
Leaf-stage heat map of differentially expressed genes in different metabolisms. (**a**) Candidate genes implicated in normal leaf development; (**b**) Genes involved in cell wall construction; **(c)** Genes involved in lignin and flavonoid synthesis; (**d**) Genes involved in synthesis of varied defensive chemicals.

During the developmental process, leaves go through photosynthetic apparatus formation, chlorophyll accumulation, cell wall hardening, and secondary metabolic transition. A number of genes that have been reported as gene-specific for leaf development were identified in the *Hevea* leaf transcriptome (Supplementary Table S5). A plant-specific lateral organ boundaries (LOB)-domain gene, *AS2*, is required for normal leaf development that induces adaxial cell fate and represses *KNOX* gene expression^17^. Here, all six LOB-domain genes (with slight variations) along with a mitotic spindle checkpoint protein MAD2-like gene^18^ were expressed mainly in developing leaves (stages I to III), but at low or very low levels in mature leaves (IV) (Fig. 3a), indicating the importance of cell division and cell expansion in the early stages of *Hevea* leaf development. In contrast, the expressions of FBPase (fructose-1,6-bisphosphatase I), SBPase (sedoheptulose-bisphosphatase) and SSII (starch synthase II) genes increased gradually with consecutive leaf stages, and peaked in mature leaves (Fig. 3a), indicating an enhancing efficiency of CO_2_ fixation with the process of leaf maturation. Sucrose is one of the main metabolic products of plant photosynthesis, as well as the most common form of carbohydrate transported from source leaves to various sink organs including the developing leaves. In the specific case of *Hevea*, sucrose represents the key precursor of natural rubber produced in its laticifers. The relative contributions of sucrose synthase and invertase to sucrose cleavage vary with plant species and stages of leaf development^19^,^20^,^21^. In soybean^21^, sucrose synthase accounts for nearly all sucrose cleavage in the youngest leaves, and nearly half of this activity in the other stages of leaf development, including the mature leaves. In *Hevea*, all three sucrose synthase (SUS) genes were more actively expressed in developing leaves than in mature leaves (Fig. 3a), suggesting an overarching importance of sucrose synthase in the sink leaves. In addition, a Snakin-1 protein (SN1), one homologue of which has been reported to be active against pathogens in potato^22^, was predominantly expressed at the early stages of *Hevea* leaf development (Fig. 3a), suggesting the involvement of this protein in defense mechanisms of young *Hevea* leaves.

### Regulation of cyanogenic glycoside metabolism in relation to leaf defense

**Figure 4:**
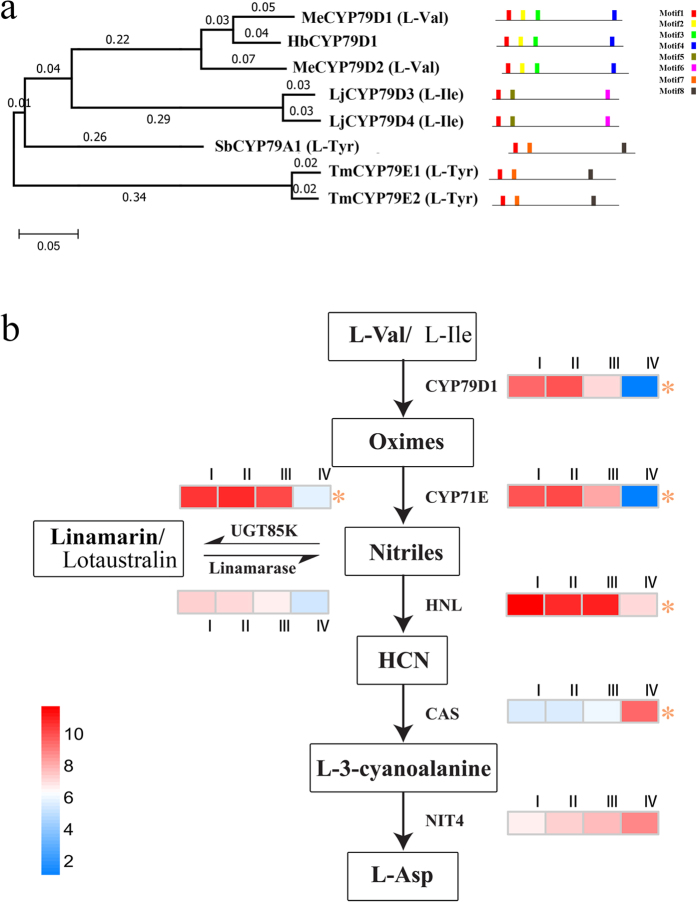
Cyanogenic glucoside metabolic pathway in *Hevea* leaves and the genes involved. (**a**) Phylogenetic relationship and motif composition of CYP79 proteins from different cyanogenic plants. Phylogenetic tree (left panel) constructed using MEGA6 with the neighbor-joining method (1000 bootstrap replicates). Schematic representation of conserved motifs (right panel) detected using MEME. Protein accessions: MeCYP79D1, GI5915822; MeCYP79D2, GI75312213; LjCYP79D3, GI75290560; LjCYP79D4, GI75290559; SbCYP79A1, GI5915822; TmCYP79E1, GI7672519; TmCYP79E2, GI7672521; (**b**) Expression profile of genes involved in the cyanogenic glucoside metabolic pathway of *Hevea* leaves.

*Hevea* is a typical cyanogenic plant, and the high cyanogenic capacity (CNc) in young leaves endows its tolerance to herbivores^23^,^24^. In-depth transcriptome sequencing and assembly allowed us to identify all the potential genes responsible for metabolism of cyanogenic glycoside (CGs) in *Hevea* leaves (Supplementary Table S6). The *Hevea* leaf CYP79D1 protein was phylogenetically clustered with the two *M. esculenta* L-Valine type homologues, and shared the same conserved motifs (Fig. 4a). Therefore, it is reasonable to predict that this *Hevea* protein catalyzes the first step in the biosynthesis of CGs, with linamarin as the major and lotaustralin the minor forms in *Hevea* leaves as its cassava homologues do^8^. In cassava, the CYP79D1/2 proteins use L-valine and L-isoleucine as substrates, respectively, to synthesize linamarin and lotaustralin. This *CYP79D1* gene and the other two downstream genes (*CYP71E* and *UGT85K*) for CGs synthesis were all abundantly expressed in immature leaves (stages I to III), but mature leaves showed low expression (stage IV) (Fig. 4b). Meanwhile, gene expressions of linamarase and hydroxynitrilelyase (HNL) that are responsible for hydrogen cyanide liberation were also much higher in young leaves than in mature leaves (Fig. 4b). These results correspond well to a higher cyanogenic potential (CNp) and CNc observed in young *Hevea* leaves than in mature ones^2^,^25^. Higher CNc is beneficial to the defense of young leaves against herbivores but may be adverse to resistance against pathogens; this could contribute to the susceptibility of young *Hevea* leaves to several economically important leaf diseases, including the devastating SALB (*Microcyclus ulei*)^25^, and the secondary leaf fall (*Oidium/Colletotrichum*) that afflicts the rubber plantations in Southeast Asia^26^,^27^.

CGs were reported to be a likely source of buffering nitrogen and glucose in *Hevea* bark, and has been implicated in rubber yield^2^. Compared to developing leaves, mature leaves showed much higher expression for the genes concerned with cyanide detoxification and utilization: one β-cyanoalanine synthase (CAS) and one β-cyanoalanine hydratase/nitrilase (NIT4A) (Fig. 4b). The results in the present study indicate that the CGs accumulated during early stages of leaf development could act as a nitrogen and carbon reserve exploited in metabolism of mature leaves. At the same time, the enhanced capability of cyanide detoxification and utilization in mature leaves could likely benefit disease resistance through decreased CNc, along with other resistance factors that make mature leaves completely resistant to SALB^1^ and the *Oidium/Colletotrichum* leaf fall^28^.

### Cell wall construction and lignin formation

In mature leaves, the thickened structure containing lignin acts as an effective physical barrier for pathogen attacks. In comparison, to accommodate cell division and expansion during the early stages of leaf development, the primary cell wall is thin, flexible and extensible. The genes involved in cell wall construction and lignin biosynthesis were identified in the *Hevea* leaf transcriptome (Supplementary Table S7). The transcript abundance of five fasciclin-like arabinogalactan proteins (FLAs) and one leucine-rich extensin (LRX) were up-regulated in young leaves (Fig. 3b). FLAs play important roles in the integrity and elasticity of plant cell wall matrix^29^, whereas LRXs are involved in cell wall assembly^30^. The abundant expressions of these genes together with three cellulose synthases (CESAs) at leaf stages I to III (Fig. 3b) are conductive to rapid cell wall construction during *Hevea* leaf development. Besides the proteins involved in cell wall construction, a variety of agents mediating cell wall loosening^31^, including the expansins (EXPs), xyloglucan endotransglycosylase/hydrolases (XTHs), and endo-1, 4-β-glucanase (EGase), also showed higher expressions at early phases of *Hevea* leaf development (Fig. 3b), implying that the molecular architecture was adjusted to the specific requirement of cell division and expansion.

Lignin is mainly deposited in the walls of secondarily thickened plant cells, having both structural and physiological functions^32^. In *Hevea*, leaves at stages I to III are almost free of lignin, and the accumulation of lignin coincides with the onset of leaf hardening and increased resistance to *M. ulei*^1^. The phenylpropanoid pathway is responsible for lignin synthesis. We identified six genes for this pathway in the *Hevea* leaf transcriptome, including two phenylalanine ammonia-lyases (PALs), one cinnamate 4-hydroxylase (C4H), two 4-coumarate-CoA ligases (4CLs), and one cinnamyl alcohol dehydrogenase (CAD) (Supplementary Table S7). All the deduced proteins had >85% alignment identity with their homologues from three other Euphorbiaceae species (*Ricinus communis*, *Manihot esculenta* and *Jatropha curcas*). CAD deoxygenizes the cinnamyl aldehydes to form the corresponding cinnamyl alcohols, the immediate building blocks of lignin (monolignols). Of the six lignin biosynthetic pathway genes, only *CAD* showed abundant and higher expressions in later leaf stages (III and IV), consistent with the commencing of lignin accumulation in *Hevea* leaves. The *CAD* gene was also expressed at early leaf stages (I and II) although to lesser levels as reported in developing leaves of tea plant^33^ and melon^34^, indicating the multiple functions of CADs in plants.

### Flavonoid biosynthetic pathway with emphasis on anthocyanin biosynthesis

Flavanoids are a large class of secondary products with multiple biological functions, including antioxidant activity as part of stress responses^35^. The coppery red leaf is a distinctively developmental initiation for *Hevea* leaves, and prompts us to investigate the synthesis of anthocyanin pigments, the most conspicuous class of flavanoids. Seven genes (families) with complete protein-coding regions were identified for the flavanoid pathway in the *Hevea* leaf transcriptome, *viz*. chalcone synthase (CHS), chalconeisomerase (ChaI), flavonoid 3-hydroxylase (F3′H), flavonoid 3′, 5′-hydroxylase (F3'5′H), dihydroflavonol-4-reductase (DFR), anthocyanidin synthase (ANS), and flavonoid 3-O-glucosyltransferase (FGT) (Supplementary Table S7). Differential patterns of expression were observed for these genes during the four stages of *Hevea* leaf development (Fig. 3c). The two upstream gene families, *CHS* and *ChaI*, showed abundant but little variance in expression throughout the four consecutive leaf stages, the pattern of which agrees with their functions in catalyzing the early steps of flavanoid synthesis, and determining the total amounts of flavanoids in different leaf stages^35^,^36^. *F3*′*H* and *F3'5*′*H* genes revealed contrasting patterns of expression in the four leaf stages, with an increasing trend for the former but a decreasing one for the latter, indicating a change in their relative importance in flavonoid biosynthesis during *Hevea* leaf development. Interestingly, all the genes involved in converting the colorless dihydroflavonols to the colored anthocyanins, *DRF*, *ANS* and *FGT*, were much higher expressed in early stages of leaf development than in the later stages. These results are consistent with the accumulation of anthocyanin pigments at the early stage of *Hevea* leaf development, and correspond well to the color change of *Hevea* leaves at four developmental stages (Fig. 1a). The colorful anthocyanins are thought to play a defense role in the delayed greening of young leaves by offsetting their structural tenderness^35^.

### Synergy among highly variable defensive components

Besides the above cyanogenic glycoside and flavonoids, varied defensive components, including phenolics, proteinase inhibitors (PIs) and lectins, contribute synergistically to efficient resistance to herbivores or pathogens in higher plants. Chitinases (CHIs) are usually considered to play a role in plant defense against pathogens, but are also involved in plant growth and development^37^. In *Arabidopsis thaliana* seedlings, mutation of a chitinase gene, *AtCTL1*, results in overproduction of ethylene and aberrant cell shapes^38^. In this study, we identified four chitinase genes (Supplementary Table S7). Although all four genes were expressed throughout the four stages of leaf development, their expression levels were much higher in developing leaves (stages I to III) than in mature ones (stage IV) (Fig. 3d), suggesting the relative importance of these genes in young leaves in either defensive or developmental roles. Similarly, several other defense-related genes, including five pathogenesis-related thaumatin superfamily proteins (PRTPs), a proteinase inhibitor 1 (PI1), and a polyphenol oxidase (PPO), also showed higher expression levels in young leaves (Fig. 3d). In contrast, a plant lectin (PL) gene was much higher expressed in mature leaves than in developing leaves, suggesting this kind of storage carbohydrate-binding proteins might take on defense roles in mature *Hevea* leaves by interfering with normal digestion and nutrient assimilation of plant-eating insects or by their antifungal properties as in other plants^39^. To determine the possibility of the above transcripts being induced by biotic stress in developing leaves, we inspected the expression levels of several indicator genes as reported in salicylic acid (SA) signaling pathway, i.e. regulatory protein NPR1, transcription factor TGA (TGA), and pathogenesis-related protein 1 (PR1)^40^. None of these genes showed a significant variance in expression levels among the four stages of leaves, ruling out the induction by biotic stress in leaf stages.

### qPCR validation of differential gene expression

**Figure 5:**
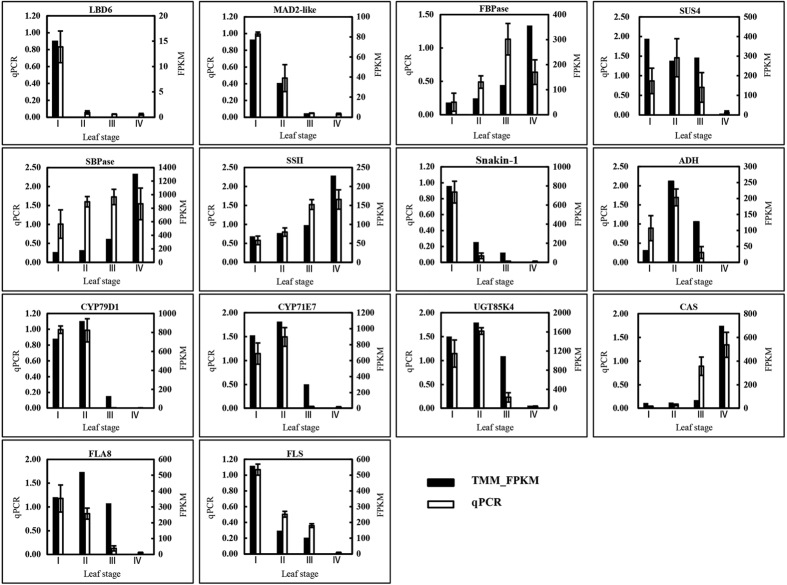
Expression of 14 selected genes as determined by qPCR in comparison to the RNA-seq results. The qPCR values for each gene are means ± SD of three biological replica. LBD, LOB Domain Protein; MAD2-like, mitotic spindle checkpoint protein MAD2-like; FBPase, fructose-1,6-bisphosphatase; SUS, sucrose synthase; SBPase, sedoheptulose-1,7-bisphosphatase; SSII, starch synthase isoform II; ADH, alcohol dehydrogenase; CYP79D1, cytochrome P450 family CYP79D1; CYP71E7, cytochrome P450 family CYP71E7; UGT85K4, UDP-glucosyltransferase; CAS, β-cyano-alanine synthase; FLA8, fasciclin-like arabinogalactan protein 8; FLS, flavonol synthase.

We validated the results of differential gene expression analysis obtained from RNA-seq data by quantitative reverse transcriptase PCR (qPCR). A total of 14 genes were selected from the above genes characterized for various metabolic pathways. The qPCR analyses were performed in the three biological replicates of leaf samples at different developmental stages from 15 *Hevea* trees. Of the 14 selected genes, 13 showed similar expression patterns in the qPCR analysis as observed from RNA-seq data (Fig. 5). The statistical analysis also showed very good correlation (r = 0.836) between the two types of analysis.

## Concluding Remarks

In this paper, we used a deep transcriptome sequencing to profile the molecular events occurring during the process of leaf development in *Hevea brasiliensis*. A series of candidate genes marking the developmental and defensive characteristics of expanding and mature leaves were found to be highly expressed at distinct leaf stages. Apparently, young and mature *Hevea* leaves exploit different defense strategies, with young leaves mainly using ‘chemical’ (defensive metabolites) but mature leaves mainly using ‘structural’ (cell wall thickness and lignification) means. Of particular interest, the developing *Hevea* leaves exploit their higher capacity for CGs synthesis and cyanide liberation to resist herbivores, and the mature leaves mainly exploit physical structure (e.g. through lignification) to defend against the leaf diseases, but utilize CGs as a nitrogen/carbon reserve instead. In remains to be seen whether the key candidate genes implicated in cyanogenic metabolism and other defensive metabolites are differentially expressed among *Hevea* cultivars or germplasms with differing resistance to herbivores and pathogens. The results of such kind of work will help engineer disease-tolerant or -resistant *Hevea* breeding materials. In addition, the giant leaf transcriptome data generated here will serve as the foundation to a systems biology approach in studying the dynamics of leaf development and defense in *Hevea* as well as in other tropical tree species.

## Materials and Methods

### Plant materials

The leaf samples in four developmental stages, i.e. bronze (I), color-change (II), pale-green (III) and bright green (mature) (IV) (Fig. 1a), were harvested in three replicates (five trees per replicate), from 10-year-old mature *Hevea* trees of the cultivar Reyan7-33-97 planted at the experimental plantation of the Rubber Research Institute, Chinese Academy of Tropical Agricultural Sciences (Danzhou, Hainan, China). The leaf development from bronze to bright green in Reyan7-33-97 lasts a period of ~20d, with 8 to 9d being bronze and 4 to 6d being color-change or pale-green. Prior to leaf harvest, the trees had been tapped every three days for two years. Collected leaves were kept in liquid nitrogen and brought to the laboratory for immediate RNA extraction as previously described^41^.

### cDNA library construction and sequencing

Four Illumina leaf cDNA libraries were prepared using the Illumina Truseq RNA sample preparation kit following the manufacturer’s instructions. The fragmented RNA was primed with N6 random hexamers to synthesize the first-strand and second-strand cDNAs. The paired-end adaptor-ligated fragments were then selected using agarose gel electrophoresis and amplified by PCR. Paired-end sequencing of the cDNA libraries was completed on the Illumina HiSeq2000 system.

### *De novo* transcriptome assembly and functional annotation

Quality estimation of the sequencing reads was conducted by FastQC (http://www.bioinformatics.bbsrc.ac.uk/projects/fastqc/). The raw sequencing reads were filtered using customized Perl scripts to remove the adaptor sequences, trim 3′ end low-quality sequence (<Q20), and discard the reads with lengths shorter than 35 bp. The resulting high-quality clean reads were those with more than 90% bases with high phred scores (≥Q20).

The clean paired-end reads were assembled using the Trinity program^42^ with the following parameters: Inchworm, -K -L 25; Chrysalis, min_glue 2 -min_iso_ratio 0.05 -glue_factor 0.05 -min 200. The assembly artifacts were then filtered out using BLASTX against the protein sequences of 26 eudicot species downloaded from the Phytozome v.10.0 database^43^ with the e-value cutoff set at 1e-2 following the methods described by Yang and Smith^15^. The paired-end reads were mapped to the *de novo* transcriptome by Bowtie2. The abundance of each transcript isoform was then estimated using RSEM^44^. The isoforms with the highest expression level in each Trinity subcomponent were pooled and then filtered for chimeras^15^ and non-plant proteins to serve as the reference transcriptome.

The reference transcriptome was annotated by a search against the databases of the NCBI Non redundant (Nr), Swiss-Prot, Phytozome v.10.0^43^, eukaryotic Orthologous (KOG) and Plant Transcription Factor^45^ using the BLASTX program (E-value ≤ 1e-5). We integrated the annotation information by a priority order of Nr, Swiss-Prot, Phytozome V10.0 and KOG database. Different transcript isoforms with the same annotation were clustered as a unigene. The Blast2GO program^46^ was used to obtain the Gene Ontology (GO) term annotations for the unigenes, and GO-terms classification was conducted on the basis of Nr annotations. Pathway annotation information was assigned using the BBH (bi-directional best hit) method of the Kyoto Encyclopedia of Genes and Genomes [KEGG] Automatic Annotation Server (KAAS) online tool^47^. To further identify the genes in the cell wall and those involved in phenylpropanoid metabolism, the deduced polypeptide sequences were annotated using the Mercator pipeline^48^ and classified into MapMan functional plant categories^49^.

### Gene expression profile and other bioinformatics analysis

Paired-end reads mapped by Bowtie2 were used for quantifying transcript abundance by RSEM software^44^. The differentially-expressed genes (DEGs) (P-value ≤ 0.01, FDR < 0.01, fold change > 2) were then identified by the ‘edgeR’ package^50^ with default parameters. The heatmap display of TMM (Trimmed Mean of M-values) normalized FPKM (Fragments Per Kilobase of transcript per Million fragments mapped) was complemented by the ‘pheatmap’ package^51^. We used the agriGO program^52^ with Fisher’s exact test to perform gene ontology enrichment analysis (P-value ≤ 0.01) and the KEGG Orthology Based Annotation System (KOBAS 2.0)^53^ with default settings to identify statistically significant enriched pathways (FDR < 0.05).

Multiple alignments of the amino acid sequences of the *Hevea* leaf CYP79D1 protein and the CYP79 proteins from other species were conducted by ClustalW^54^, and were then subjected to phylogenetic analysis using the MEGA 6 software^55^ with neighbor-joining method and 1000 bootstrap replicates. The conserved motifs were identified by the MEME suit with default parameters (http://meme-suite.org/).

### qRCR validation

Quantitative reverse transcriptase PCR (qPCR) were performed as describe by Tang *et al*.^41^. Specific primers for respective DEGs (Supplementary Table S8) were designed by Primer5, and all of the amplified fragments were sequenced for target verification. The qPCR reaction was performed on the Light Cycler 2.0 System (Roche Diagnostics, Penzberg, Germany) using the SYBR Green premix kit (TaKaRa) according to the manufacturer’s instructions. All reactions had three biological replicates, each with two technical repeats. *YLS8* was used as the internal control for gene expression analyses as described previously^56^. The efficiency of each primer pair was estimated as ranging from 1.841 and 2.001. The relative abundance of transcripts was calculated using the LightCycler Relative Quantification Software. The correlations between the results of qPCR and RNA-seq data analyses were determined for 14 selected genes by R scripts using Spearman’s rank correlation coefficient.

## Additional Information

**Accession codes**: All the clean reads of four libraries have been deposited in the Sequence Read Archive at the NCBI database with the accessions SRR3136185, SRR3136188, SRR3136190 and SRR3136192.

**How to cite this article**: Fang, Y. *et al. De novo* Transcriptome Analysis Reveals Distinct Defense Mechanisms by Young and Mature Leaves of *Hevea brasiliensis* (Para Rubber Tree). *Sci. Rep*. **6**, 33151; doi: 10.1038/srep33151 (2016).

## Supplementary Material

Supplementary Information

Supplementary Table S1

Supplementary Table S2

Supplementary Table S3

## Figures and Tables

**Table 1:** Assembly and annotation statistics of the *Hevea* leaf transcriptome.

Total clean reads	62,609,749
Trinity Assembly	
Number of contigs	199,472
Non-redundant Contigs	104,137
Chimeras contigs	338
Contigs encoding non-plant proteins	5,003
Final unique transcripts	98,796
Final transcripts N50 (bp)	936
Annotation	
Nr	29,592
Swiss-Prot	19,216
Phytozome V10	34,982
KOG	27,509
