# Supplementary material for: De novo Transcriptome Analysis Reveals Distinct Defense Mechanisms by Young and Mature Leaves of Hevea brasiliensis (Para Rubber Tree)
Source: Sci Rep. 2016 Sep 13;6:33151. doi: 10.1038/srep33151 (PMC5020607; doi:10.1038/srep33151)
Supplement: Supplementary Information [file srep33151-s1.pdf]

De novo Transcriptome Analysis Reveals Distinct Defense Mechanisms by Young and Mature Leaves of *Hevea brasiliensis* (Para Rubber Tree)

Yongjun Fang[1,+], Hailiang Mei[2,3,+], Binhui Zhou[1], Xiaohu Xiao[1], Meng Yang[2], Yacheng Huang[1], Xiangyu Long[1], Songnian Hu[2*], Chaorong Tang[1*]

1 Rubber Research Institute, Chinese Academy of Tropical Agricultural Sciences, Danzhou 571737, Hainan, China

2 CAS Key Laboratory of Genome Sciences and Information, Beijing Institute of Genomics, Chinese Academy of Sciences, Beijing 100101, China

3 University of Chinese Academy of Sciences, Beijing 100101, China

[+] Co-first authors.

[*]Correspondence should be addressed to CRT (chaorongtang@126.com) or SNH (husn@big.ac.cn).

Supplementary Figures:

Supplementary Figure S1. Estimation of sequencing depth of *Hevea* leaf RNA-seq data. (A) N50 of transcripts assembled from different sequencing depth; (B) Saturation curves for detected genes and ratio of unique mapped reads.

Supplementary Tables:

Supplementary Table S1. Differentially expressed genes between leaf development stage I and stage II

Supplementary Table S2. Differentially expressed genes between leaf development stage II and stage III

Supplementary Table S3. Differentially expressed genes between leaf development stages III and IV

Supplementary Table S4. GO term and KEGG pathway enrichment for the DEGs of clusters 1 and 4 in Fig.3b

Supplementary Table S5. Candidate genes specific for *Hevea* leaf development

Supplementary Table S6. Genes for cyanogenic glycoside metabolism in *Hevea* leaves

Supplementary Table S7. Genes related to biosynthesis of cell wall structure, phenylpropanoid, flavonoids and various defensive proteins

Supplementary Table S8. Primers used for quantitative PCR analysis

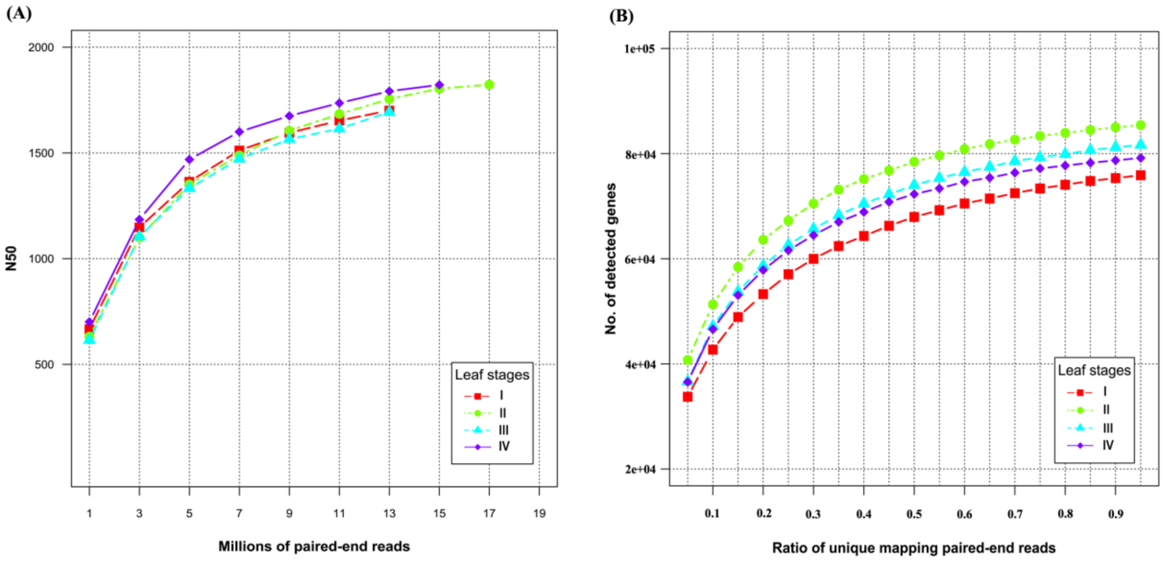

Supplementary Figure S1. Estimation of sequencing depth of *Hevea* leaf RNA-seq data. (A) N50 of transcripts assembled from different sequencing depth; **(B)** Saturation curves for detected genes and ratio of unique mapped reads.

Table S4. GO term and KEGG pathway enrichment for the DEGs of clusters 1 and 4 in Fig.3b

DEGs of cluster 1

Gene Ontology Term	GO ID	P-value	FDR
phosphonate transport	GO:0015716	8.30E-13	6.30E-10
transmembrane transport	GO:0055085	1.30E-09	5.30E-07
nitrate transport	GO:0015706	7.10E-08	1.50E-05
oxidation reduction	GO:0055114	1.10E-08	3.30E-06
establishment of localization	GO:0051234	6.90E-08	1.50E-05
carbohydrate transport	GO:0008643	4.30E-05	4.40E-03
**KEGG pathway**	**Pathway ID**	**P-value**	**FDR**
Thiamine metabolism	rcu00730	0.0011342	0.0483709
ABC transporters	rcu02010	0.0016874	0.0483709

DEGs of cluster 4

Gene Ontology Term	GO ID	P-value	FDR
anatomical structure development	GO:0048856	2.9E-70	2.1E-68
multicellular organismal development	GO:0007275	3.5E-56	2.5E-54
cell growth	GO:0016049	1.8E-22	1.3E-20
cell differentiation	GO:0030154	6.5E-17	4.6E-15
signal transduction	GO:0007165	0.00021	0.015
cell wall	GO:0005618	9.6E-34	3.80E-32
**KEGG pathway**	**Pathway ID**	**P-value**	**FDR**
Starch and sucrose metabolism	rcu00500	2.88E-17	2.418E-15
Phenylpropanoid biosynthesis	rcu00940	5.04E-09	1.775E-07
Amino sugar and nucleotide sugar metabolism	rcu00520	6.70E-06	1.41E-04
Cutin, suberine and wax biosynthesis	rcu00073	4.07E-04	4.89E-03
Cyanoamino acid metabolism	rcu00460	7.27E-04	7.64E-03
Brassinosteroid biosynthesis	rcu00905	9.65E-04	9.00E-03

Table S5. Candidate genes specific for *Hevea* leaf development

**Gene**	**Transcript ID**	**Length (aa)**	**Accession No.**	**Species**	**Length (aa)**	**Identity**
LBD	comp53490_c0_seq1	221	XP012078919	*Jc*	218	97%
	comp50990_c0_seq2	222	XP011027000	*Pe*	216	82%
	comp46441_c0_seq1	165	XP002300051	*Pt*	161	88%
	comp83196_c0_seq1	170	XP002528321	*Rc*	176	78%
	comp50448_c1_seq2	244	XP002525255	*Rc*	246	80%
	comp29657_c0_seq1	218	XP002527499	*Rc*	236	74%
MAD2-like	comp47245_c0_seq1	207	XP002514967	*Rc*	207	97%
FBPase	comp48935_c0_seq2	410	XP002527886	*Rc*	410	92%
SUS	comp56967_c0_seq4	806	AGM14949	*Hb*	806	99%
	comp57152_c0_seq2	841	AGM14946	*Hb*	830	93%
	comp33842_c1_seq1	675	AGM14951	*Hb*	904	99%
SBPase	comp53396_c0_seq1	389	XP002530415	*Rc*	389	91%
SSII	comp52212_c0_seq1	751	ABV25894	*Me*	751	87%
	comp46105_c0_seq6	600	XP012082557	*Jc*	602	83%
	comp28935_c0_seq2	640	ABV25893	*Me*	633	86%
SN1	comp18190_c0_seq1	88	KHN42338	*Gs*	90	77%

Abbreviations: LBD, LOBDomain Protein; MAD2-like, mitotic spindle checkpoint protein MAD2-like; FBPase, fructose-1,6-bisphosphatase; SUS, sucrose synthase; SBPase, sedoheptulose-1,7- bisphosphatase; SSII, starch synthase isoform II; SN1, Snakin-1; *Jc*, *Jatropha curcas; Pe, Populus euphratica; Pt, Populus trichocarpa; Rc, Ricinus communis; Hb, Hevea brasiliensis; Me, Manihot esculenta; Gs, Glycine soja*.

Table S6. Genes for cyanogenic glycoside metabolism in *Hevea* leaves

Gene	Transcripts ID	Length (aa)	Accession No.	Species	Length (aa)	Identity
CYP79D1	comp53519_c0_seq1	539	Q9M7B8	*Me*	542	90%
CYP71E	comp58894_c0_seq1	511	Q6XQ14	*Me*	511	95%
UGT85K	comp48977_c0_seq1	484	AEO45782	*Me*	483	87%
Linamarase	comp41664_c0_seq4	490	AAB22162	*Me*	531	46%
HNL	comp56879_c1_seq11	256	CAA11219	*Me*	258	81%
CAS	comp54463_c0_seq2	367	ADO78230	*Me*	370	92%
NIT4A	comp42921_c0_seq1	361	XP_002523857	*Rc*	351	91%

Abbreviations: CYP79D1, cytochrome P450 CYP79D1;CYP71E, cytochrome P450CYP71E; UGT85K, ; HNL, hydroxynitrilelyase; CAS, β-cyanoalanine synthase; NIT4A, nitrilase 4A;NIT2, nitrilase2; NIT4B, nitrilase 4B.

Table S7. Genes related to biosynthesis of cell wall structure, phenylpropanoid, flavonoids and various defensive proteins

**Gene name**	**Unigene ID**	**Length (bp)**	**Species**	**GenBank Accession**	**Protein length**	**Blastp Identity**
FLA	comp46773_c0_seq1	1,552	*Jc*	XP012081483	408	82.00%
	comp58975_c0_seq1	2,135	*Md*	XP008343059	425	79.00%
	comp54946_c1_seq7	1,936	*Jc*	XP012074266	466	92.00%
	comp45988_c0_seq2	1,450	*Jc*	XP012080695	260	83.00%
	comp53402_c1_seq1	1,498	*Jc*	XP012071008	404	88.00%
LRX	comp53276_c0_seq2	1,684	*Rc*	XP002514194	414	82.00%
EXP	comp55567_c1_seq1	1,144	*Jc*	XP012088304	266	91.00%
	comp46727_c0_seq1	1,574	*Jc*	XP012074731	281	94.00%
XTH	comp29740_c0_seq1	1,168	*Jc*	XP012078987	288	91.00%
	comp53197_c0_seq4	1,299	*Jc*	XP012091402	299	91.00%
	comp19199_c0_seq1	1,171	*Cs*	XP006487657	319	91.00%
CESA	comp58235_c0_seq1	3,939	*Rc*	XP002532166	1085	90.00%
	comp58389_c0_seq1	4,082	*Jc*	XP012075293	1097	95.00%
	comp57588_c0_seq2	3,836	*Tc*	XP007013842	1085	92.00%
	comp58127_c0_seq1	3,427	*Jc*	XP012091811	981	93.00%
EGase	comp53608_c0_seq1	1,664	*Hb*	AKH03678	497	99.00%
	comp57167_c0_seq2	2,374	*Rc*	XP002510516	523	94.00%
	comp52710_c0_seq1	1,716	*Hb*	AKH03675	494	99.00%
	comp53510_c0_seq1	2,180	*Pe*	XP011041962	510	89.00%
	comp53591_c0_seq2	2,430	*Hb*	AKH03679	620	98.00%
	comp44986_c0_seq1	1,900	*Hb*	AKH03682	521	99.00%
PAL	comp54077_c0_seq1	2,412	*Rc*	XP002514077	688	88.00%
	comp56139_c0_seq1	2,618	*Me*	AAK60275	712	95.00%
C4H	comp50629_c0_seq1	2,073	*Ca*	ACR10242	505	92.00%
4CL	comp56080_c0_seq1	2,473	*Jc*	XP012067426	543	89.00%
	comp29392_c0_seq1	1,998	*Jc*	XP012064722	542	87.00%
CAD	comp54109_c0_seq1	1,507	*Hb*	ADU64756	357	97.00%
CHS	comp54261_c0_seq3	1,623	*Gh*	ABS52573	389	90.00%
	comp722155_c0_seq1	771	*Rc*	XP002529257	387	89.00%
ChaI	comp50577_c1_seq6	1,073	*Gh*	ADG27840	209	87.00%
	comp37701_c0_seq2	1,176	*Pt*	XP002315258	223	78.00%
F3'H	comp39926_c0_seq2	1,885	*Rc*	XP002532045	505	82.00%
F3'5'H	comp125327_c0_seq1	1,718	*Tc*	XP007015254	528	82.00%
DFR	comp18727_c0_seq1	1,286	*Vr*	AGJ70142	337	79.00%
ANS	comp58956_c0_seq1	1,760	*Tc*	ADD51356.1	354	86.00%
FGT	comp23206_c0_seq1	1,893	*Me*	Q40287	487	79.00%
	comp57496_c0_seq1	1,780	*Pt*	XP002312969	477	54.00%
CHI	comp49564_c0_seq1	1,147	*Vv*	CAC14015	325	81.00%
	comp48288_c0_seq1	1,408	*Rc*	XP002519197	324	90.00%
	comp58874_c0_seq1	3,049	*Rc*	XP002515664	321	84.00%
	comp28713_c0_seq1	1,193	*Hb*	P23472	311	99.00%
PI1	comp50948_c0_seq1	667	*Hb*	Q6XNP7	70	100.00%
PPO	comp43128_c0_seq1	1,861	*Hb*	AHF20575	600	99.00%

PL	comp45247_c0_seq2	809	*Rc*	XP002321809	177	54.00%
PRTP	comp46369_c0_seq1	1,596	*Hb*	ALE20175	327	99.00%
	comp51275_c0_seq1	1,344	*Tc*	XP007036794	275	87.00%
	comp54107_c0_seq1	1,136	*Jc*	XP012074976	240	92.00%
	comp82086_c0_seq1	915	*Jc*	XP012082655	261	81.00%
	comp38129_c0_seq1	1,799	*Tc*	XP007035697	160	66.00%
NPR1	comp57405_c0_seq2	2,958	*Rc*	XP002520549	590	86.00%
	comp55401_c2_seq6	1,951	*Jc*	XP012076961	585	85.00%
TGA	comp57164_c1_seq4	2,559	*Pe*	XP011027648	362	76.00%
	comp37385_c0_seq1	1,997	*Jc*	XP012075186	374	91.00%
PR1	comp50118_c0_seq1	827	*Pe*	XP011016555	159	83.00%
	comp48757_c0_seq1	982	*Jc*	XP012082230	194	58.00%

Abbreviations:FLA, fasciclin-like arabinogalactan protein; LRX, leucine-rich extensin; EXP, expansin; XTH, xyloglucanendotransglycosylase/hydrolases; CESA, cellulose synthase; EGases, endo-1,4-β-glucanases; PAL, phenylalanine ammonia-lyase; C4H, cinnamate 4-hydroxylase; 4CL, 4-coumarate-CoA ligase; CAD, cinnamyl alcohol dehydrogenase; CHS, chalcone synthase; ChaI, chalconeisomerase; F3'H, flavonoid 3-hydroxylase; F3'5'H, flavonoid 3',5'-hydroxylase; DFR, dihydroflavonol-4-reductase; ANS, anthocyanidin synthase; FGT, flavonoid 3-O-glucosyltransferase; CHI, chitinase; PI1, protease inhibitor 1; PPO, polyphenol oxidase; PL, plant lectin;PRTP, pathogenesis-related thaumatin superfamily protein; NPR1, regulatory protein NPR1; TGA, transcrtption factor TGA; PR1, pathogenesis-related protein 1; *Ca*, *Canarium album*; *Gh*, *Gossypium hirsutum*; *Md*, *Malus domestica*; *Tc*, *Theobroma cacao*; *Vr*, *Vitis rotundifolia*; *Vv*, *Vitis vinifera*.

Table S8. Primers used for quantitative PCR analysis

Gene	Transcripts_ID	Forward Primer	Reverse primer
flavonol synthase	comp46686_c0_seq1	5'-CCTCCGACGGATTTTGAAGTG-3'	5'-CACCACTCGTTACATCACACAAC-3'
alcohol dehydrogenase	comp34385_c0_seq2	5'-GCTTGCCAAATCTGATGTCAAGT-3'	5'-GCTCCCAGGAAGTGAGAGTAG-3'
mitotic spindle checkpoint protein MAD2-like	comp47245_c0_seq1	5'-CGTCAGATTGCTTCGAGTATTACT-3'	5'-CCAGAGTGTCAACCTTGTGT-3'
fructose-1,6-bisphosphatase	comp48935_c0_seq2	5'-GACCAGAAGAAGCTTGACGTC-3'	5'-CAAGAGTGTTGTCGTCACCAA-3'
UGT85K4	comp48977_c0_seq1	5'-GCATAATCATAGACGCTTAATTCGCA-3'	5'-TTCTTTTAGCTTGGCAATTAGCTCAA-3'
starch synthase isoform II	comp52212_c0_seq1	5'-GTGAGTGCTTTTCCACGTAGTC-3'	5'-TCACCTGGCCTGCTTATTCT-3'
sedoheptulose-1,7-bisphosphatase	comp53396_c0_seq1	5'-TAACTGCTTATCTAGTCTCTTACAGTC-3'	5'-TGAACTTGATTTCAGATTCCTGG-3'
LOB domain protein 6	comp53490_c0_seq1	5'-CAAGGAAATATATGTTTGCCATGCC-3'	5'-CTTCAAATTGTAAATAATGTTGTTGTCCA-3'
beta-cyanoalanine synthase	comp54463_c0_seq2	5'-CAGACTGTTTCAGGACATTTGTAG-3'	5'-CTCATTTGTAAGTTTCAGAGTTCATG-3'
sucrose synthase 4	comp56967_c0_seq4	5'-TCGATGGAAGTGTCAATGGCAAC-3'	5'-GGCAGTGGACTTTCAGAAACTCG-3'
CYP71E7	comp58894_c0_seq1	5'-GTGCCTCTTTCAAAATCACCAAG-3'	5'-GAAGTGTGGAACTAGTAGTGGAAC-3'
Fasciclin-like arabinogalactan protein 8 precursor	comp58975_c0_seq1	5'-GTGGACTATGGCTAAGCATGAT-3'	5'-GGAAAGATCGACCACCGTTA-3'
Snakin-1	comp18190_c0_seq1	5’-GCTATAGGGACAAGAAGAACTCAAAG-3’	5’-AGAGCAAATCCAAATCCACAGA-3’
CYP79D1	comp53519_c0_seq1	5’-TTGAAAGAGAGTGATGAATCTGAGT-3’	5’-CTCAATAACAAGCAGTCTGTAAGGT-3’
